# Nuclear actin filaments – a historical perspective

**DOI:** 10.1080/19491034.2024.2320656

**Published:** 2024-02-21

**Authors:** Maria Kristha Fernandez, Molika Sinha, Mia Zidan, Malte Renz

**Affiliations:** Gynecologic Oncology Division, School of Medicine Stanford University, Palo Alto, CA, USA

**Keywords:** nuclear actin filaments, nucleocytoplasmic shuttling, Biomechanics of cell nucleus, DNA damage repair, signaling and gene expression

## Abstract

The view on nuclear filaments formed by non-skeletal β-actin has significantly changed over the decades. Initially, filamentous actin was observed in amphibian oocyte nuclei and only under specific cell stress conditions in mammalian cell nuclei. Improved labeling and imaging technologies have permitted insights into a transient but microscopically apparent filament network that is relevant for chromatin organization, biomechanics of the mammalian cell nucleus, gene expression, and DNA damage repair. Here, we will provide a historical perspective on the developing insight into nuclear actin filaments.

## Introduction

**Figure 1.**
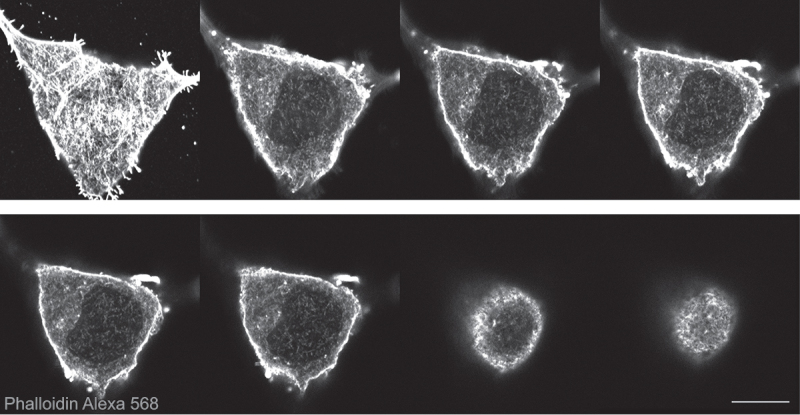
Breast cancer cells (BT-20) showing nuclear actin filaments stained with phalloidin Alexa 568. Representative slides of a z-stack. Scale bar 10 μm.

Actin is one of the most conserved proteins throughout the evolution of eukaryotes, and one of the most abundant proteins in eukaryotic cells. It exists in monomeric globular form (G-actin) and in polymeric filamentous forms of different lengths (F-actin). There is a high concentration of actin in the cytoplasm and a low concentration in the nucleus, forming a steep concentration gradient across the nuclear envelope. Cytoplasmic actin is involved in a multitude of functions including cell motility, cytokinesis, organelle movement, and cell signaling. While initial reports on actin’s existence in the cell nucleus were met with doubt as probably artifactual, increasingly more functions of actin in the cell nucleus have been recognized over the last few decades, functions of actin in its monomeric form but also its polymeric form. In steady state, low actin concentration in the nucleus – and the contrasting high abundance in the cytoplasm – may have hampered insights into the existence and function of nuclear actin. Here, we focused only on findings regarding filamentous actin in the cell nucleus of non-muscle cells (Figure 1). We will account for the findings chronologically and organized in thematic sections and thereby try to provide a historical perspective on nuclear actin filaments.

## Amphibian oocyte nuclei, biomechanics, and actin export

In the 1960s, structural changes in the cell nuclei of amphibian embryos and oocytes were noted after the exposure to actinomycin-D (Act-D), an antibiotic that binds DNA and inhibits DNA-dependent RNA-synthesis. In these studies, Act-D was used for its DNA binding capacity to study secondary structures of nucleic acids and its effects on differentiation. Jones et al. described ‘loose groupings of uniform threads’ with a diameter of 200 Å, 0.5–2 μm long and apparently composed of subfilaments within cell nuclei of the frog *Rana pipiens* embryos after ActD exposure. Jones interpreted those threads as uncoiled chromosomes [1,2]. Lane noted in maturing oocytes of the newt *Triturus viridescens* after Act-D exposure fibrillar bodies composed of bundled filaments of 50–70 Å in diameter and up to 64 μm in length. Although of unknown composition, Lane showed that the filaments were Feulgen-negative and not likely related to chromosomes [3]. While these first experiments employed Act-D, subsequent publications realized that actin is a constituent of the amphibian oocyte nucleus even under native conditions. In the late 1970s, Merriam and Clark characterized actin as a major component of the high-speed supernatant fraction of *Xenopus* egg extracts. In the warmth, this component formed a gel which over time contracted to a small aggregate [4,5]. Clark and Rosenbaum showed electron micrographs of an actin filament network in hand-isolated nuclei of *Xenopus* eggs that bound rabbit skeletal muscle myosin subfragment-1 [6]. In the 1980s and 90s, actin filaments were seen in intact frog *Rana temporaria* oocytes in stage 6 using electron microscopy [7], and later again in stages 3 and 6 [8]. For the *in vitro* reconstruction of cell nuclei, demembranated sperm was added to *Xenopus* egg extract. The addition of rhodamine-labeled actin to the egg extracts helped reveal the development of a nuclear actin filament network in such reconstituted cell nuclei [9]. Upon addition of the actin depolymerizing, Latrunculin A nuclear assembly was distorted. Field emission scanning electron microscopy was used to characterize nuclear actin filaments in frog oocytes and their contacts to nuclear pore complexes and nuclear organelles [10,11]. Similar contacts at the inner nuclear membrane of stage 6 *Xenopus* oocyte nuclei were described using cryo-scanning electron microscopy (EM) [12].

In 2006, Bohnsack et al. [13] discovered that in *Xenopus* oocytes exportin-6 (XPO-6) is a developmentally regulated transport protein that is absent in the initial stages of oocyte development but increasingly synthesized from meiotic maturation onwards. With increasing expression of XPO-6, the nuclear actin concentration decreased, and the filament network disappeared. The authors suggested that the sponge-like nuclear actin filament network was required for additional mechanical support for frog egg oocyte nuclei which are 100,000 times larger than somatic cell nuclei [14]. Later, another study reported that depolymerization of nuclear actin with cytochalasin D and latrunculin A resulted in chromosome condensation [15]. Following up on these results, Brangwynne and Feric presented experimental and theoretical evidence that in cell nuclei greater than 10 μm in size gravity is an increasingly potent force and that the nuclear actin filament network in the large *Xenopus* egg nuclei was required to prevent nucleoli and histone locus bodies from undergoing gravitational sedimentation and fusion [16].

In line with these results, *Xenopus* blastula, but not *Xenopus* gastrula, required nuclear actin filaments for the stability of their cell nuclei and proper alignment of chromosomes on the mitotic spindle [17]. Nuclei assembling in *Xenopus* egg extracts reportedly showed a bilobed shape and assumed a more round shape upon the addition of lamin A (LMNA), which is absent from *Xenopus* eggs [18].

## Cell stress response in mammalian cell nuclei, and actin import

About a decade after the first reports of filamentous actin structures in amphibian oocyte nuclei in the 1970s and 80s, various cell stresses were reported to induce nuclear actin filaments in mammalian cells. Dimethyl sulfoxide (DMSO) was the first and most studied cell stressor in the 1980s. Visualization was initially achieved by electron microscopy (EM) and later by fluorescence microscopy.

In 1978, Fukui et al. presented the first EM images of nuclear actin filaments in the interphase cell nucleus of the slime mold *Dictyostelium* after 30-min exposure to 10% DMSO [19]. The filaments bound rabbit skeletal muscle heavy meromyosin. This binding could be reversed in the presence of Mg^2+^ and ATP. In 1979, Fukui showed that DMSO also triggered nuclear actin filaments in *Amoeba* and HeLa cells [20] and in 1982 in the ciliate *Tetrahymena* [21]. Nuclear actin filament formation was reversible with DMSO removal [22]. In 1980, two groups, Sanger et al. [23] and Osborn et al. [24], presented similar findings of reversibility in mammalian epithelial kidney cells, Ptk2, with dissolution of cytoplasmic filaments and formation of nuclear filaments and the reversal of those findings 1–2 hours after removal of DMSO. Both groups discussed a possible exchange of actin between the cytoplasm and cell nucleus. Filament formation was inhibited by ATP-inhibitors [25]. In another follow-up study, Sanger et al. injected fluorescently labeled actin into the cytoplasm and found that it was incorporated into actin stress fibers. The addition of DMSO resulted in the reversible breakdown of these actin stress fibers and the appearance of fluorescent inclusions in the cell nucleus, demonstrating reversible translocation of cytoplasmic actin into the cell nucleus [26].

In 1985 and 1986, Welch et al. [27] and Iida et al. demonstrated that the reversible loss of cytoplasmic stress fibers and the development of intranuclear paracrystal-like actin structures could also be induced by heat shock [28]. In a follow-up study, a year later from the cell biology and biophysics groups from Tokyo, the 21 kDa actin-binding and severing protein cofilin was found to be a component of the nuclear actin rods that developed with DMSO exposure, heat shock, and salt buffers. The actin rods were not stained with phalloidin but with cofilin antibodies. Thus, these nuclear actin rods were assumed to be different from stress fibers and likely right-handed helices [29]. In further follow-up studies, cofilin’s nuclear localization signal (NLS) was identified which mediated the translocation of actin and cofilin upon heat shock [30]. A different group identified a nuclear export signal (NES) important for the cofilin export via CRM1 (chromosomal maintenance 1, later renamed exportin-1 or XPO-1) [31]. Furthermore, the actin depolymerizing agent Latrunculin B and ATP-depletion was described to induce cofilin-dependent translocation of actin into the cell nucleus of mast cells [32].

### Role in degeneration and apoptosis

While nuclear actin filaments were noted in a stress response to DMSO, heat shock, etc., they were found to be reversible. In a few publications, however, a possible role of nuclear actin filaments in degeneration and apoptosis was suggested. In 1983, Radley and Haller reported the existence of bundles of parallel filaments in the cell nucleus of degenerating mouse megakaryocytes after the exposure to 5-fluorouracil. The bundles were 7 nm in diameter and morphologically similar to the described filaments upon exposure to DMSO or heat shock [33]. In the early 2000s, the human leukemia cell line HL-60 undergoing hyperthermia showed thin actin bundles in early apoptotic nuclei. It was suggested that nuclear actin filaments could be involved in chromatin rearrangement during apoptosis [34]. Another study reported nuclear actin filaments in HL-60 and the lymphoblast cells K-562 after the exposure to etoposide and doxorubicin, as assessed by phalloidin staining and EM imaging using gold labeling [35]. In a follow-up study, the authors described the colocalization of nuclear F-actin and SATB1 (special AT-rich sequence-binding protein) during apoptosis induced by geldanamycin in MCF-7 cells [36].

## Nucleocytoplasmic compartmentalization, actin-binding proteins and labeling strategies

Monomeric globular actin is 42 kDa in size and of compact structure. It has no classic NLS but may pass through nuclear pores by diffusion even in complex with small proteins. Actin comprises two conserved nuclear export signals (NES) [37]. The known active nuclear import mechanism is the co-shuttling of cofilin-actin mediated by importin-9 (IMP-9) [38]; the active export of the complex profilin-actin is mediated by exportin-6 (XPO-6) [39]. Additional nuclear export by XPO-1 (or CRM1) has been reported via an unknown mechanism [40]. Actin appears to be transported as a monomer in both directions; therefore, transport is limited by the available G-actin pool.

In 2006, using fluorescence recovery after photobleaching (FRAP) of GFP-labeled actin and R62D actin, an actin mutant that cannot polymerize, as well as antibody- and FITC-labeled actin, the Hendzel group described that approximately 20% of nuclear actin exists in polymeric form and turns over rapidly [41]. In 2013, Vartiainen and coworkers examined import and export rates of GFP-actin in relationship to the nucleocytoplasmic fluorescence intensity ratio using FRAP assays. They found that, in steady state, nuclear actin levels are determined by export competent actin monomers. The creation of an export incompetent actin pool by binding to nuclear complexes retains actin in the cell nucleus and makes it available for nuclear functions, so the authors [42]. In the same year, Henderson and colleagues used FRAP analyses of GFP-actin and reported increased rates of nuclear actin import during G1/S phase arrest using thymidine and DNA replication stress with hydroxyurea. The authors also noted under the above circumstances an increased import of Rac1 (Ras-related C3 botulinum toxin substrate 1) and IQGAP1 (IQ motif containing GTPase activating protein 1), both stimulators of actin polymerization [43].

Several studies on intracellular shuttling of actin-binding proteins noted a shift in actin’s nucleocytoplasmic compartmentalization and an increase in nuclear polymerized actin. In 1999, the N-terminal fragment of supervillin, which contains its NLS and actin binding sites, was expressed in mammalian cells and found to induce nuclear actin filaments stainable with phalloidin and lamin A/C, however, resistant to latrunculin A [44]. Similarly, overexpression of the C-terminal region of myosin 16b with its NLS resulted in nuclear actin filaments that could be co-stained with profilin antibodies and phalloidin and delayed S-phase progression [45]. Conversely, knockdown of Arp4, which is predominantly located in the cell nucleus, was reported to trigger nuclear actin filament formation [46]. Emerin increased actin polymerization in the presence of capping proteins by 4- to 12-fold and was suggested to contribute to an actin-based network at the inner nuclear membrane [47].

In the context of actin’s nucleocytoplasmic shuttling, the steep concentration gradient across the nuclear envelope and its propensity to polymerize once a critical concentration threshold are surpassed, the difficulties in labeling nuclear actin and the various efforts that have been made to overcome these difficulties should be mentioned. Initial work on cofilin-actin rods suggested that nuclear actin does not exist in the phalloidin-stainable polymerized form. Different actin conformations were explored. Antibodies specific to actin-dimers and low-number polymers were reported [48,49]. Mullins and coworkers examined the effects of various probes employing actin-binding domains. They suggested that LifeAct [50], which is comprised of the first 17 amino acids of Abp 140p (actin-binding protein 140) found in yeast [51], and Utrophin (Utr261), which comprises two calponin homology domains, induced and stabilized nuclear actin filaments that can be stained with phalloidin. Instead, the authors suggested a truncated utrophin probe tagged to a nuclear localization sequence, Utr230-EN, and the RPEL domain from MRTF-A (or MAL) that binds monomeric actin [52]. Utr230-EN, which comprises only one intact calponin homology domain, was capable of binding filaments in the cytoplasm and recognized punctae in the nucleus. Subsequently, Feng and coworkers reported that LifeAct and Utr230 induced distinct actin assemblies in the cell nucleus: LifeAct induced filaments and Utr230 punctae and aggregates [53]. In 2015, Grosse and coworkers tagged an NLS to the commercially available actin-chromobody-TagGFP [54]. The actin chromobody comprised a variable heavy domain of heavy-chain antibodies (VHH) found in the family Camelidae, which includes Llamas and Alpaca. These VHH or nanobodies were originally described in 1993 [55]. In their 2015 publication, Grosse et al. noted that a potential stabilization of filamentous actin was limiting the use of LifeAct [54].

In 2022, Uyeda and colleagues reported the visualization of nuclear actin filaments with actin-GFP (instead of the commonly employed sequence of FP-actin) that could not be stained with LifeAct, phalloidin, or actin antibodies but co-stained with cofilin-GFP [56]. The group around Knöll tagged an NLS to the following probes and thereby targeted them to the cell nucleus: (i) wild-type flag-tagged β-actin, the two polymer-stabilizing actin mutants (ii) S14C and (iii) G15S (with the latter being deficient in interacting with cofilin) as well as the polymerization-deficient actin mutant (iv) R62D. The resulting overexpression of β-actin in the cell nucleus led to nuclear actin filament formation that could be stained with phalloidin and LifeAct-GFP and imaged using electron microscopy [57]. Hozák and co-workers reported that the overexpression of YFP-NLS-actin resulted in only a minority of cells (up to 5%) in nuclear filaments which showed colocalization only with cofilin antibodies and displayed delayed mitosis [58].

## Virus assembly and transport

In 1988, Volkman presented evidence that nuclear actin filaments were involved in the proper nucleocapsid formation of the baculovirus *Autographa californica* nuclear polyhedrosis virus, a large double-stranded DNA virus of lepidopteran insects. Cytochalasin D (CytD) disrupted its nucleocapsid formation, and phalloidin stained the nuclei of the infected host cells [59]. In a follow-up study, Volkman and colleagues reported that actin filaments were found locally in the cell nucleus in the virogenic stroma at the time of exponential virus production [60]. In another follow-up, the authors intended to provide further evidence since nuclear F-actin had never been described for any other virus. They created cell clones of *Spodoptera frugiperda* expressing CytD-resistant actin and showed differential sensitivity of the virus production to CytD compared to wild-type cells [61]. The group of Chen from Wuhan described a WASP homologue, the HearNPV ORF 2(HA2), in the nuclear polyhedrosis viruses that may nucleate nuclear actin filaments in the presence of Arp2/3 [62]. In a follow-up study, the same group characterized actin nucleation by the virus protein further and defined a critical actin-binding pocket [63]. A different study showed that the baculovirus protein VP80 was involved in the polar transport of the nucleocapsid to the nuclear periphery [64]. More recent studies with engineered *Autographa californica* nuclear polyhedrosis virus were confirmatory [65].

Based on single particle tracking, Forest et al. postulated in 2005, an active intranuclear transport for herpesvirus capsids, and suggested that this transport was dependent on nuclear actin and possibly myosin [66]. In 2006, Enquist and coworkers reported that nuclear actin filaments were required for the viral capsid assembly of the two herpes viruses, pseudorabies virus (PRV) and HSV-1, and speculated that the viral capsids may travel along the filaments in a myosin V dependent fashion [67]. Later, Enquist et al. reopened the question as to how nuclear herpesvirus capsids move in the host nucleus based on results using LifeAct-GFP and phalloidin [68]. In 2016, Coen and coworkers showed evidence using stable cells expressing LifeAct-GFP that human CMV but not HSV, that is, beta- and not alpha-herpes viruses induced nuclear actin filaments in the host cell that promote capsid movement to the nuclear periphery and its nuclear egress [69].

In the tobamovirus Turnip vein clearing virus, a plant virus, a new nuclear stage in the infection cycle was identified, which involved the interaction with nuclear filamentous actin and chromatin [70].

## Biomechanics of the mammalian cell nucleus

Recognizing a biomechanical role in large amphibian oocyte nuclei and viral capsid transport, increasingly more biomechanical functions of nuclear actin filaments were described in mammalian cells as well. In the early 1990s, Haskin et al. reported that in the osteosarcoma cell line MG-63 intranuclear actin inclusions formed, while cytoplasmic actin stress fibers disappeared during the 20-min exposure to 4.0 MPa hydrostatic pressure [71]. Actin rods were visualized by EM and fluorescence microscopy in the cytoplasm and nucleus of *Dictyostelium* spores. It has been hypothesized that those rods may absorb pressure and help maintain the shape of the spores [72].

In 2005, Ellenberg and coworkers demonstrated that in the large starfish oocytes, during the first meiotic division, the microtubule spindle was not sufficiently large to capture the chromosomes. After nuclear envelope breakdown, the authors visualized a nuclear actin meshwork. This actin ’fishnet’ was employed for long-range motion to bring the chromosomes into the capture distance of the microtubule spindle [73]. Based on *in vitro* experiments, emerin, a lamin-interacting protein, was proposed to promote a nuclear actin cortical network likely contributing to the stability of the cell nucleus [47]. With the actin chromobody targeted to the cell nucleus (nAcTagGFP-NLS), the Grosse group described transient nuclear actin filament formation in NIH3T3 upon cell spreading and growth on fibronectin. This filament formation could be suppressed by the knockdown of (i) Sun 1 or Sun2, components of the LINC complex, (ii) lamin A/C, and (iii) emerin [54]. The same group found using the same actin-chromobody probe cell-cycle specific transient nuclear actin polymerization at the mitotic exit that facilitated volume expansion of the daughter cell nuclei and chromosome decondensation [74]. In a follow-up study, α-actinin 4 was identified as a critical component of the nuclear actin filaments that formed during mitotic exit [75]. Mao and coworkers reported that short nuclear actin filaments in G1 were required to maintain CENP-A (centromere protein A) levels at the centromere, important for the correct segregation of chromosomes during mitosis [76]. Recently, employing Utr262 and phalloidin, F-actin was visualized during mitosis of *Zebrafish* embryos. It was hypothesized that F-actin plays a role in proper mitotic progression, assisting with nuclear envelope breakdown, chromosome congression, and spindle assembly in *Zebrafish* embryos [77].

## Signaling and gene expression

It is well established that monomeric globular actin is associated with all three RNA polymerases in the cell nucleus and acts in concert with nuclear myosin I to drive transcription [78–81]. In 2007, Grummt and coworkers presented biochemical evidence that also polymerized nuclear actin in concert with myosin I increased transcription [82]. In another study examining the reprogramming of transplanted somatic nuclei into amphibian oocytes, filamentous actin appeared to be critical for the reactivation of the pluripotency gene *Oct4* [83], likely mediated by the nuclear actin-binding protein Wave1 (WASP family verprolin-homologous protein) [84]. A more recent study reported the formation of nuclear actin filaments after somatic nuclear transfer into mouse embryonic cells [85].

In 2013, Grosse and colleagues demonstrated that serum stimulation of serum-starved cells resulted in transient, minute-long nuclear actin filament formation detected by LifeAct-GFP-NLS and phalloidin. The nuclear actin filament formation was dependent on the formin mDia (mammalian diaphanous). Just as in the cytoplasm, actin polymerization in the cell nucleus released MAL (megakaryocytic acute leukemia protein), also called MRTF-A (myocardin-related transcription factor A), a serum response factor (SRF), which in turn activated transcriptional activity [86]. In follow-up studies, the authors described that the stimulation of G protein-coupled receptors with ligands such as thrombin, lysophosphatidic acid (LPA), and ATP resulted in transient calcium spikes within the cell nucleus that preceded transient nuclear actin filament formation [87,88]. In 2020, the Sun group described RNA polymerase II clustering with the nucleus upon serum stimulation requiring nuclear actin filaments and WASP [89]. In 2014, Jaffrey and coworkers showed that MICAL-2 (microtubule associated monooxygenase, calponin and LIM domain containing 2), an atypical actin-regulatory protein, oxidized actin at methionine 44 and thereby depolymerized nuclear filamentous actin which in turn increased nuclear MRTF-A levels. The precise mechanism remained unknown since the increase of G-actin should increase G-actin binding to MRTF-A, its subsequent export via XPO-1 (or CRM1) and thus decrease the MRTF-A concentration and activity in the cell nucleus [90]. Another regulator of nuclear actin concentration, RASSF1A (Ras association domain family isoform A), a tumor suppressor gene, was described to support the binding of XPO-6 to a RAN GTPase and thereby facilitated the nuclear export of the actin-profilin complex. Loss of RASSF1A was reported to decrease MRTF-A transcriptional activity, likely through an increase in nuclear G-actin concentration [91].

Contrasting reports on increased transcription, Lanerolle and coworkers in 2016 stabilized nuclear F-actin by expressing (i) the actin mutant V163M-α-actin, (ii) a supervillin fragment (1–1010 aa), and (iii) YFP-labeled nuclear targeted actin in mammalian cells. The α-actin mutant is a point mutation occurring in intranuclear rod myopathy whose hallmark is nuclear actin filament formation. All of these constructs resulted in reduced monomeric nuclear actin and reduced RNA-polymerase II activity and transcription [92]. Furthermore, the authors found that heat shock induced cofilin-actin rods which in turn reduced RNA polymerase II activity [93].

In 2019, the Fackler and Grosse laboratories reported that Ca^2+^-regulated nuclear actin polymerization identified by nuclear targeted LifeAct-GFP allowed CD4+ helper cells to swiftly convert T-cell receptor signals into effector functions and cytokine expression [94]. Another study found that the artificial accumulation of actin in the cell nucleus and its polymerization resulted in the nuclear accumulation and transcriptional activity of β-catenin [95].

The heterochromatin remodeling complex SWI/SNF (switch/sucrose non-fermentable) was shown to bind purified F-actin *in vitro*, mediated by its ATPase Brg1 [96]. Mammalian cells that expressed Flag-tagged NLS-actin and grew on a stiff extracellular matrix, i.e., on stretch displayed nuclear actin filaments that showed an association to the endogenous SWI/SNF complex. The binding of actin filaments to the SWI/SNF complex prevented in turn the binding of ARID1A (AT-rich interactive domain-containing protein 1A) to YAP/TAZ (yes-associated protein and transcriptional coactivator with PDZ-binding motif). Thus, free YAP/TAZ could bind to DNA-binding transcription factors. When grown on a soft substrate, no nuclear actin filaments were formed, and only the loss of ARID1A disinhibited YAP/TAZ activity [97]. Another study linked nuclear F-actin visualized with LifeAct-FP to an increase in liver cancer metastases. Interfering with the TCA (tricarboxylic acid) cycle by TFAM (transcription factor A, mitochondrial) deficiency resulted in increased malonyl-CoA which in turn increased mDia malonylation, its nuclear translocation and the induction of nuclear actin filaments, so the authors [98].

Long-range movement of chromatin sites in yeast for transcription was reported to involve ARP, Hsp90, myosin motors, and nuclear filamentous actin which was visualized with the actin chromobody [99]. A role of nuclear filamentous actin for long-range chromosomal motion had been implicated previously [100–102]. A role in nuclear mRNA transport was also discussed. It was suggested that nuclear actin filaments may be involved in restricting immature mRNA to the cell nucleus [103,104].

## DNA damage repair

The earliest reports of nuclear actin filaments in amphibia oocyte nuclei and mammalian cells notably employed potentially DNA affecting agents. More recently, a role of nuclear actin filaments in DNA damage repair has come increasingly more into focus as detailed below.

The overexpression of cofilin down-regulated key components of the DNA double-strand break repair systems including Rad51 (radiation-sensitive protein 51), Rad52, Ku70/Ku80 (‘Ku’ from the surname of the Japanese patient, 70/80 kDa in size). Thereby, the radiosensitivity of the lung cancer cell line NCI-H1299 increased, as reported by Keng and coworkers in 2005 [105]. In 2012, Hendzel and colleagues presented *in vitro* evidence that purified Ku70/80 binds to polymerized actin. The Ku heterodimer is known to recognize and bind to the ends of DNA double strand break and is required for the error-prone DNA double strand repair by non-homologous end-joining (NHEJ). The authors found in cells that (i) the non-polymerizing mutant actin R62D targeted to the cell nucleus and (ii) the depolymerization with cytochalasin D changed the retention of Ku80 at DNA damage sites, suggesting that filamentous actin was required for stabilization of the Ku heterodimer at the DNA damage site and thereby proper DNA double-strand break repair [106]. Later, in 2015, Mullins and coworkers found that genotoxins including UV light and 0.01% methyl methanesulfonate (MMS) induced nuclear actin filaments which were visualized with Utr230-3×NLS and phalloidin [107]. Formin-2 and Spire-1/Spire-2 nucleated actin filaments after DNA damage. The experimental decrease in Formin-2 or importin-9 increased the number of DNA double-strand breaks. Similarly, in a study on cumulus-enclosed mouse oocytes, DNA damage induced by bleomycin triggered nuclear actin filaments that could be detected using phalloidin [108]. Chiolo and coworkers connected nuclear F-actin to chromatin dynamics and heterochromatin repair. Heterochromatin double-strand breaks in *Drosophila* cells undergo homologous recombination in the nuclear periphery. The group showed in 2018 that directed motion of heterochromatin was mediated by nuclear actin filaments and nuclear myosins which were recruited to the repair site [109]. In 2018, Gautier and colleagues showed that nuclear actin, ARP2/3 and WASP were recruited to damaged chromatin undergoing homology-directed repair, which in contrast to non-homologous end-joining repair according to their findings required enhanced motion. The authors demonstrated that nuclear actin polymerization was required for the long-range migration of a subset of double-strand breaks into discrete nuclear clusters [110].

Cesare and group described in 2020 that nuclear F-actin participated in the replication stress response during S-phase. Nuclear F-actin increased with an increasing dose of aphidicolin which is a reversible inhibitor of DNA replication and blocks cells in early S phase. Nuclear actin was visualized with phalloidin as well as with actin-FP and actin chromobody both targeted to the cell nucleus. Nuclear actin was regulated by the ATR-dependent (ataxia telangiectasia and Rad3-related protein) activation of mTORC1 (mammalian target of rapamycin complex 1) and nucleated through IQGAP1, WASP, and ARP2/3. Nuclear F-actin increased the nuclear volume and sphericity and, in concert with myosin II, increased the mobility of the stress-replication foci toward the nuclear periphery, as per the author’s findings. Furthermore, in mouse xenografts using the osteosarcoma cell line U2-OS stably expressing the nuclear actin chromobody, the authors showed with *in vivo* imaging nuclear actin polymerization after replication-stress inducing chemotherapy with carboplatin and hydroxyurea [111]. Another study showed that nuclear F-actin formed a network for nuclear bodies, which is essential for DNA damage repair in promyelocytic leukemia (PML) cells. This network could not form when prelamin A was overexpressed. Actin was visualized using the nuclear actin chromobody [112]. Recently, in 2023, Blanpain and coworkers demonstrated that the small GTPase RHOJ regulated the epithelial-to-mesenchymal transition and was associated with chemotherapy resistance by enhancing the response to replicative stress and activating DNA damage response that allowed tumor cells to repair chemotherapy-induced DNA damage. RHOJ interacted with proteins that regulate nuclear actin. The inhibition of nuclear actin polymerization sensitized cells to chemotherapy-induced cell death [113]. In another study from the Chiolo and Lopes labs that had not been peer-reviewed at the time of this review, nuclear F-actin was found to be involved in replication fork plasticity in S-phase by slowing replication fork progression and inducing fork reversal. Nuclear actin filaments increased in size and number upon genotoxic treatment including etoposide, as visualized with nuclear actin chromobodies. If nuclear F-actin was inhibited, reduced recruitment of Rad51 and SMARCAL1 (SWI/SNF-related matrix-associated actin-dependent regulator of chromatin subfamily A-like protein 1) to the DNA was noted; instead, increased PrimPol (primase and DNA directed polymerase) resulted in chromosomal instability and decreased cellular resistance to replication stress [114].

## Conclusion

**Figure 2.**
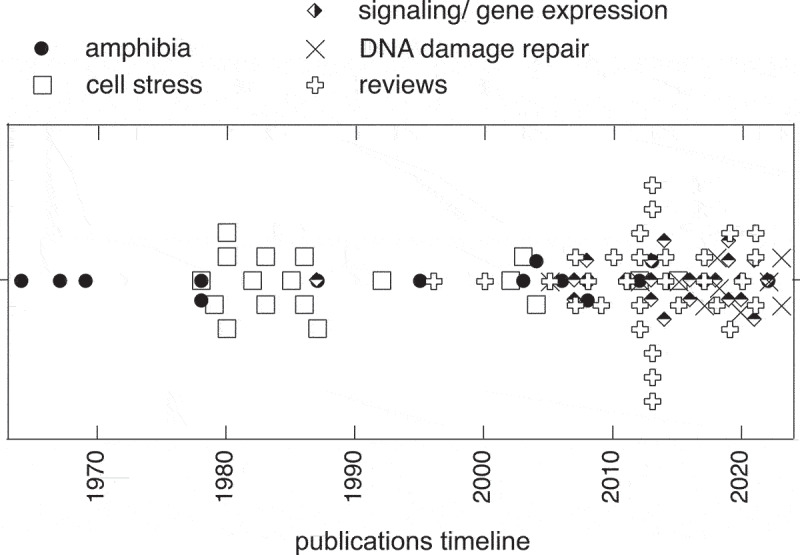
Timeline of primary research data publications and review publications centered on nuclear actin filaments.

In this historical perspective, we outlined the chronology of the findings related to nuclear actin filaments in thematic sections (Figure 2). We believe that this approach helps provide perspective. For example, it illustrates how the initial findings of a nuclear actin filament system in frog egg nuclei led to the characterization of the main thus far known nuclear actin export mediated by XPO-6, while the initial studies on cell stressors in mammalian cells resulted in the characterization of its major active importer IMP-9. Actin’s nucleocytoplasmic shuttling, which maintains a steep concentration gradient across the nuclear envelope, provides context for the choice of nuclear actin probes and its potential implications. In fact, temporary changes in actin’s nucleocytoplasmic shuttling appear to be critical for the temporary increase in nuclear actin concentration and the temporary emergence of nuclear actin filaments that has been reported over decades. The temporarity, the steep concentration gradient with a very low actin concentration in the cell nucleus, and the low concentration threshold for actin to polymerize may have hampered insight into nuclear actin filaments; however, improved actin probes and imaging techniques have resulted in the more recent findings of the transient minute-long formation of a nuclear actin network that is involved in signal transduction, chromatin transport, and DNA damage repair.

## Data Availability

Data sharing is not applicable to this article as no new data were created or analyzed in this study.

## References

[1] Jones KW, Elsdale TR. The effects of actinomycin D on the ultrastructure of the nucleus of the amphibian embryonic cell. J Cell Bio. 1964;21(2):245–13. doi: 10.1083/jcb.21.2.245 14159027 PMC2106436

[2] Jones KW. The induction of paracrystalline thread-complexes in the nuclei of amphibian cells by actinomycin D and other DNA-binding antibiotics. J Ultrastruct Res. 1967;18(1):71–84. doi: 10.1016/S0022-5320(67)80232-6 4164905

[3] Lane NJ. Intranuclear fibrillar bodies in actinomycin D-treated oocytes. J Cell Bio. 1969;40(1):286–91. doi: 10.1083/jcb.40.1.286 5812427 PMC2107599

[4] Clark TG, Merriam RW. Actin in xenopus oocytes. J Cell Bio. 1978;77(2):427–38. doi: 10.1083/jcb.77.2.427 565781 PMC2110058

[5] Merriam RW, Clark TG. Actin in xenopus oocytes. II. Intracellular distribution and polymerizability. J Cell Bio. 1978;77(2):439–447. doi: 10.1083/jcb.77.2.439 565782 PMC2110040

[6] Clark TG, Rosenbaum JL. An actin filament matrix in hand-isolated nuclei of X. laevis oocytes. Cell. 1979;18(4):1101–1108. doi: 10.1016/0092-8674(79)90223-X 574804

[7] Parfenov VN, Galaktionov KI. [Intranuclear actin microfilaments in the oocytes of the common frog]. Tsitologiia. 1987;29(2):142–149. 2437673

[8] Parfenov VN, Davis DS, Pochukalina GN, et al. Nuclear actin filaments and their topological changes in frog oocytes. Exp Cell Res. 1995;217(2):385–394. doi: 10.1006/excr.1995.1101 7698240

[9] Krauss SW, Chen C, Penman S, et al. Nuclear actin and protein 4.1: essential interactions during nuclear assembly in vitro. Proc Natl Acad Sci U S A. 2003;100(19):10752–7. doi: 10.1073/pnas.1934680100 12960380 PMC196875

[10] Kiseleva E, Drummond SP, Goldberg MW, et al. Actin- and protein-4.1-containing filaments link nuclear pore complexes to subnuclear organelles in xenopus oocyte nuclei. J Cell Sci. 2004;117(12):2481–2490. doi: 10.1242/jcs.01098 15128868

[11] Morozova KN, Kiseleva EV. [Changes in xenopus oocyte nucleus and cytoplasm organization after actin filaments depolymerization by latrunculin]. Tsitologiia. 2008;50(5):394–405. 18683585

[12] Walther P. High-resolution cryo-SEM allows direct identification of F-actin at the inner nuclear membrane of xenopus oocytes by virtue of its structural features. J Microsc. 2008;232(2):379–85. doi: 10.1111/j.1365-2818.2008.02109.x 19017237

[13] Bohnsack MT, Stüven T, Kuhn C, et al. A selective block of nuclear actin export stabilizes the giant nuclei of Xenopus oocytes. Nat Cell Biol. 2006;8(3):257–63. doi: 10.1038/ncb1357 16489345

[14] Gall JG. Exporting actin. Nat Cell Biol. 2006;8(3):205–7. doi: 10.1038/ncb0306-205 16508670

[15] Maslova A, Krasikova A. Nuclear actin depolymerization in transcriptionally active avian and amphibian oocytes leads to collapse of intranuclear structures. Nucleus. 2012;3(3):300–11. doi: 10.4161/nucl.20393 22572951 PMC3414407

[16] Feric M, Brangwynne CP. A nuclear F-actin scaffold stabilizes ribonucleoprotein droplets against gravity in large cells. Nat Cell Biol. 2013;15(10):1253–9. doi: 10.1038/ncb2830 23995731 PMC3789854

[17] Oda H, Shirai N, Ura N, et al. Chromatin tethering to the nuclear envelope by nuclear actin filaments: a novel role of the actin cytoskeleton in the xenopus blastula. Genes Cells. 2017;22(4):376–391. doi: 10.1111/gtc.12483 28318078

[18] Mishra S, Levy DL. Nuclear F-actin and Lamin a antagonistically modulate nuclear shape. J Cell Sci. 2022;135(13):135(13. doi: 10.1242/jcs.259692 PMC9377710 35665815

[19] Fukui Y. Intranuclear actin bundles induced by dimethyl sulfoxide in interphase nucleus of dictyostelium. J Cell Bio. 1978;76(1):146–57. doi: 10.1083/jcb.76.1.146 338616 PMC2109968

[20] Fukui Y, Katsumaru H. Nuclear actin bundles in Amoeba, Dictyostelium and human HeLa cells induced by dimethyl sulfoxide. Exp Cell Res. 1979;120(2):451–455. doi: 10.1016/0014-4827(79)90412-9 571346

[21] Katsumaru H, Fukui Y. In vivo identification of tetrahymena actin probed by DMSO induction of nuclear bundles. Exp Cell Res. 1982;137(2):353–63. doi: 10.1016/0014-4827(82)90036-2 6799309

[22] Fukui Y, Katsumaru H. Dynamics of nuclear actin bundle induction by dimethyl sulfoxide and factors affecting its development. J Cell Bio. 1980;84(1):131–40. doi: 10.1083/jcb.84.1.131 7188610 PMC2110527

[23] Sanger JW, Gwinn J, Sanger JM. Dissolution of cytoplasmic actin bundles and the induction of nuclear actin bundles by dimethyl sulfoxide. J Exp Zool. 1980;213(2):227–30. doi: 10.1002/jez.1402130210 6894001

[24] Osborn M, Weber K. Dimethylsulfoxide and the ionophore A23187 affect the arrangement of actin and induce nuclear actin paracrystals in PtK2 cells. Exp Cell Res. 1980;129(1):103–114. doi: 10.1016/0014-4827(80)90335-3 6775963

[25] Sanger JW, Sanger JM, Jockusch BM. Differential response of three types of actin filament bundles to depletion of cellular ATP levels. Eur J Cell Biol. 1983;31(2):197–204. 6685626

[26] Sanger JW, Sanger JM, Kreis TE, et al. Reversible translocation of cytoplasmic actin into the nucleus caused by dimethyl sulfoxide. Proc Natl Acad Sci U S A. 1980;77(9):5268–72. doi: 10.1073/pnas.77.9.5268 7001475 PMC350039

[27] Welch WJ, Suhan JP. Morphological study of the mammalian stress response: characterization of changes in cytoplasmic organelles, cytoskeleton, and nucleoli, and appearance of intranuclear actin filaments in rat fibroblasts after heat-shock treatment. J Cell Bio. 1985;101(4):1198–211. doi: 10.1083/jcb.101.4.1198 3900086 PMC2113902

[28] Iida K, Iida H, Yahara I. Heat shock induction of intranuclear actin rods in cultured mammalian cells. Exp Cell Res. 1986;165(1):207–215. doi: 10.1016/0014-4827(86)90545-8 3519257

[29] Nishida E, Iida K, Yonezawa N, et al. Cofilin is a component of intranuclear and cytoplasmic actin rods induced in cultured cells. Proc Natl Acad Sci U S A. 1987;84(15):5262–6. doi: 10.1073/pnas.84.15.5262 3474653 PMC298835

[30] Iida K, Matsumoto S, Yahara I, et al. The KKRKK sequence is involved in heat shock-induced nuclear translocation of the 18-kDa actin-binding protein, cofilin. Cell Struct Funct. 1992;17(1):39–46. doi: 10.1247/csf.17.39 1586966

[31] Munsie LN, Desmond CR, Truant R. Cofilin nuclear-cytoplasmic shuttling affects cofilin-actin rod formation during stress. J Cell Sci. 2012;125(Pt 17):3977–88. doi: 10.1242/jcs.097667 22623727

[32] Pendleton A, Pope B, Weeds A, et al. Latrunculin B or ATP depletion induces cofilin-dependent translocation of actin into nuclei of mast cells. J Biol Chem. 2003;278(16):14394–400. doi: 10.1074/jbc.M206393200 12566455

[33] Radley JM, Haller CJ. Fate of senescent megakaryocytes in the bone marrow. Br J Haematol. 1983;53(2):277–87. doi: 10.1111/j.1365-2141.1983.00201.x-i1 6821656

[34] Luchetti F, Burattini S, Ferri P, et al. Actin involvement in apoptotic chromatin changes of hemopoietic cells undergoing hyperthermia. Apoptosis. 2002;7(2):143–52. doi: 10.1023/A:1014362415047 11865198

[35] Grzanka A, Grzanka D, Orlikowska M. Fluorescence and ultrastructural localization of actin distribution patterns in the nucleus of HL-60 and K-562 cell lines treated with cytostatic drugs. Oncol Rep. 2004;11(4):765–70. doi: 10.3892/or.11.4.765 15010870

[36] Grzanka D, Kowalczyk AE, Izdebska M, et al. The interactions between SATB1 and F-actin are important for mechanisms of active cell death. Folia Histochem Cytobiol. 2015;53(2):152–61. doi: 10.5603/fhc.a2015.0018 26315726

[37] Wada A, Fukuda, M, Mishima, M, et al. Nuclear export of actin: a novel mechanism regulating the subcellular localization of a major cytoskeletal protein. EMBO J. 1998;17(6):1635–41. doi: 10.1093/emboj/17.6.1635 9501085 PMC1170511

[38] Dopie J, Skarp K-P, Kaisa Rajakylä E, et al. Active maintenance of nuclear actin by importin 9 supports transcription. Proc Natl Acad Sci U S A. 2012;109(9):E544–52. doi: 10.1073/pnas.1118880109 22323606 PMC3295300

[39] Stuven T, Hartmann E, Gorlich D. Exportin 6: a novel nuclear export receptor that is specific for profilin.actin complexes. EMBO J. 2003;22(21):5928–5940. doi: 10.1093/emboj/cdg565 14592989 PMC275422

[40] Vartiainen MK, Guettler S, Larijani B, et al. Nuclear actin regulates dynamic subcellular localization and activity of the SRF cofactor MAL. Science. 2007;316(5832):1749–1752. doi: 10.1126/science.1141084 17588931

[41] McDonald D, Carrero G, Andrin C, et al. Nucleoplasmic β-actin exists in a dynamic equilibrium between low-mobility polymeric species and rapidly diffusing populations. J Cell Bio. 2006;172(4):541–552. doi: 10.1083/jcb.200507101 16476775 PMC2063674

[42] Skarp KP, Huet G, Vartiainen MK. Steady-state nuclear actin levels are determined by export competent actin pool. Cytoskeleton (Hoboken). 2013;70(10):623–34. doi: 10.1002/cm.21116 23749625

[43] Johnson MA, Sharma M, Mok MTS, et al. Stimulation of in vivo nuclear transport dynamics of actin and its co-factors IQGAP1 and Rac1 in response to DNA replication stress. Biochim Biophys Acta. 2013;1833(10):2334–47. doi: 10.1016/j.bbamcr.2013.06.002 23770048

[44] Wulfkuhle JD, Donina IE, Stark NH, et al. Domain analysis of supervillin, an F-actin bundling plasma membrane protein with functional nuclear localization signals. J Cell Sci. 1999;112(**Pt 13**):2125–2136. doi: 10.1242/jcs.112.13.2125 10362542

[45] Cameron RS, Liu C, Mixon AS, et al. Myosin16b: the COOH-tail region directs localization to the nucleus and overexpression delays S-phase progression. Cell Motil Cytoskeleton. 2007;64(1):19–48. doi: 10.1002/cm.20162 17029291

[46] Yamazaki S, Gerhold C, Yamamoto K, et al. The Actin-Family Protein Arp4 is a novel suppressor for the formation and functions of nuclear F-Actin. Cells. 2020;9(3):758. doi: 10.3390/cells9030758 32204557 PMC7140684

[47] Holaska JM, Kowalski AK, Wilson KL, et al. Emerin caps the pointed end of actin filaments: evidence for an actin cortical network at the nuclear inner membrane. PLoS Biol. 2004;2(9):E231. doi: 10.1371/journal.pbio.0020231 15328537 PMC509406

[48] Gonsior SM, Platz S, Buchmeier S, et al. Conformational difference between nuclear and cytoplasmic actin as detected by a monoclonal antibody. J Cell Sci. 1999;112(**Pt 6**):797–809. doi: 10.1242/jcs.112.6.797 10036230

[49] Schoenenberger CA, Buchmeier S, Boerries M, et al. Conformation-specific antibodies reveal distinct actin structures in the nucleus and the cytoplasm. J Struct Biol. 2005;152(3):157–68. doi: 10.1016/j.jsb.2005.09.003 16297639

[50] Riedl J, Crevenna AH, Kessenbrock K, et al. Lifeact: a versatile marker to visualize F-actin. Nat Methods. 2008;5(7):605–7. doi: 10.1038/nmeth.1220 18536722 PMC2814344

[51] Asakura T, Sasaki T, Nagano F, et al. Isolation and characterization of a novel actin filament-binding protein from Saccharomyces cerevisiae. Oncogene. 1998;16(1):121–130. doi: 10.1038/sj.onc.1201487 9467951

[52] Belin BJ, Cimini BA, Blackburn EH, et al. Visualization of actin filaments and monomers in somatic cell nuclei. MboC. 2013;24(7):982–994. doi: 10.1091/mbc.e12-09-0685 23447706 PMC3608506

[53] Du J, Fan Y-L, Chen T-L, et al. Lifeact and Utr230 induce distinct actin assemblies in cell nuclei. Cytoskeleton (Hoboken). 2015;72(11):570–5. doi: 10.1002/cm.21262 26538385

[54] Plessner M, Melak M, Chinchilla P, et al. Nuclear F-actin formation and reorganization upon cell spreading. J Biol Chem. 2015;290(18):11209–16. doi: 10.1074/jbc.M114.627166 25759381 PMC4416828

[55] Hamers-Casterman C, Atarhouch T, Muyldermans S, et al. Naturally occurring antibodies devoid of light chains. Nature. 1993;363(6428):446–8. doi: 10.1038/363446a0 8502296

[56] Nagasaki A, Katoh K, Hoshi M, et al. Characterization of phalloidin-negative nuclear actin filaments in U2OS cells expressing cytoplasmic actin-EGFP. Genes Cells. 2022;27(5):317–330. doi: 10.1111/gtc.12930 35194888

[57] Kokai E, Beck H, Weissbach J, et al. Analysis of nuclear actin by overexpression of wild-type and actin mutant proteins. Histochem Cell Biol. 2014;141(2):123–35. doi: 10.1007/s00418-013-1151-4 24091797

[58] Kalendova A, Kalasová I, Yamazaki S, et al. Nuclear actin filaments recruit cofilin and actin-related protein 3, and their formation is connected with a mitotic block. Histochem Cell Biol. 2014;142(2):139–52. doi: 10.1007/s00418-014-1243-9 25002125 PMC4110419

[59] Volkman LE. Autographa californica MNPV nucleocapsid assembly: inhibition by cytochalasin D. Virology. 1988;163(2):547–553. doi: 10.1016/0042-6822(88)90295-4 3281373

[60] Charlton CA, Volkman LE. Sequential rearrangement and nuclear polymerization of actin in baculovirus-infected Spodoptera frugiperda cells. J Virol. 1991;65(3):1219–27. doi: 10.1128/jvi.65.3.1219-1227.1991 1995943 PMC239889

[61] Ohkawa T, Volkman LE. Nuclear F-actin is required for AcMNPV nucleocapsid morphogenesis. Virology. 1999;264(1):1–4. doi: 10.1006/viro.1999.0008 10544124

[62] Wang Q, Liang C, Song J, et al. HA2 from the helicoverpa armigera nucleopolyhedrovirus: a WASP-related protein that activates Arp2/3-induced actin filament formation. Virus Res. 2007;127(1):81–7. doi: 10.1016/j.virusres.2007.03.021 17467839

[63] Li K, Wang Y, Bai H, et al. The putative pocket protein binding site of autographa californica nucleopolyhedrovirus BV/ODV-C42 is required for virus-induced nuclear actin polymerization. J Virol. 2010;84(15):7857–68. doi: 10.1128/JVI.00174-10 20484515 PMC2897618

[64] Marek M, Merten O-W, Galibert L, et al. Baculovirus VP80 protein and the F-actin cytoskeleton interact and connect the viral replication factory with the nuclear periphery. J Virol. 2011;85(11):5350–62. doi: 10.1128/JVI.00035-11 21450830 PMC3094977

[65] Fu Y, Lin T, Liang A, et al. Effects of recombinant baculovirus AcMNPV-BmK IT on the formation of early cables and nuclear polymerization of actin in Sf9 cells. Cytotechnology. 2016;68(3):381–7. doi: 10.1007/s10616-014-9789-x 25698159 PMC4846645

[66] Forest T, Barnard S, Baines JD. Active intranuclear movement of herpesvirus capsids. Nat Cell Biol. 2005;7(4):429–31. doi: 10.1038/ncb1243 15803134

[67] Feierbach B, Piccinotti S, Bisher M, et al. Alpha-herpesvirus infection induces the formation of nuclear actin filaments. PLOS Pathog. 2006;2(8):e85. doi: 10.1371/journal.ppat.0020085 16933992 PMC1550268

[68] Bosse JB, Virding S, Thiberge SY, et al. Nuclear herpesvirus capsid motility is not dependent on F-actin. MBio. 2014;5(5):e01909–14. doi: 10.1128/mBio.01909-14 25293761 PMC4196236

[69] Wilkie AR, Lawler JL, Coen DM, et al. A Role for Nuclear F-Actin Induction in Human Cytomegalovirus Nuclear Egress. MBio. 2016;7(4). doi: 10.1128/mBio.01254-16 PMC4999551 27555312

[70] Levy A, Zheng JY, Lazarowitz SG. The tobamovirus turnip vein clearing virus 30-kilodalton movement protein localizes to novel nuclear filaments to enhance virus infection. J Virol. 2013;87(11):6428–40. doi: 10.1128/JVI.03390-12 23536678 PMC3648121

[71] Haskin CL, Athanasiou KA, Klebe R, et al. A heat-shock-like response with cytoskeletal disruption occurs following hydrostatic pressure in MG-63 osteosarcoma cells. Biochem Cell Biol. 1993;71(7–8):361–71. doi: 10.1139/o93-054 7510113

[72] Sameshima M, Kishi Y, Osumi M, et al. Novel actin cytoskeleton: actin tubules. Cell Struct Funct. 2000;25(5):291–295. doi: 10.1247/csf.25.291 11235897

[73] Lenart P, Bacher CP, Daigle N, et al. A contractile nuclear actin network drives chromosome congression in oocytes. Nature. 2005;436(7052):812–8. doi: 10.1038/nature03810 16015286

[74] Baarlink C, Plessner M, Sherrard A, et al. A transient pool of nuclear F-actin at mitotic exit controls chromatin organization. Nat Cell Biol. 2017;19(12):1389–1399. doi: 10.1038/ncb3641 29131140

[75] Krippner S, Winkelmeier J, Knerr J, et al. Postmitotic expansion of cell nuclei requires nuclear actin filament bundling by α-actinin 4. EMBO Rep. 2020;21(11):e50758. doi: 10.15252/embr.202050758 32959960 PMC7645226

[76] Liu C, Zhu R, Mao Y. Nuclear actin polymerized by mDia2 confines centromere movement during CENP-A loading. iScience. 2018;9:314–327. doi: 10.1016/j.isci.2018.10.031 30448731 PMC6240728

[77] Oda H, Sato Y, Kawashima SA, et al. Actin filaments accumulated in the nucleus remain in the vicinity of condensing chromosomes in the zebrafish early embryo. Biol Open. 2023;12(5). doi: 10.1242/bio.059783 PMC10214854 37071022

[78] Philimonenko VV, Zhao J, Iben S, et al. Nuclear actin and myosin I are required for RNA polymerase I transcription. Nat Cell Biol. 2004;6(12):1165–72. doi: 10.1038/ncb1190 15558034

[79] Hu P, Wu S, Hernandez N. A role for beta-actin in RNA polymerase III transcription. Genes Dev. 2004;18(24):3010–3015. doi: 10.1101/gad.1250804 15574586 PMC535912

[80] Kukalev A, Nord Y, Palmberg C, et al. Actin and hnRNP U cooperate for productive transcription by RNA polymerase II. Nat Struct Mol Biol. 2005;12(3):238–44. doi: 10.1038/nsmb904 15711563

[81] Percipalle P, Fomproix N, Kylberg K, et al. An actin–ribonucleoprotein interaction is involved in transcription by RNA polymerase II. Proc Natl Acad Sci U S A. 2003;100(11):6475–6480. doi: 10.1073/pnas.1131933100 12743363 PMC164471

[82] Ye J, Zhao J, Hoffmann-Rohrer U, et al. Nuclear myosin I acts in concert with polymeric actin to drive RNA polymerase I transcription. Genes Dev. 2008;22(3):322–30. doi: 10.1101/gad.455908 18230700 PMC2216692

[83] Miyamoto K, Pasque V, Jullien J, et al. Nuclear actin polymerization is required for transcriptional reprogramming of Oct4 by oocytes. Genes Dev. 2011;25(9):946–58. doi: 10.1101/gad.615211 21536734 PMC3084028

[84] Miyamoto K, Teperek M, Yusa K, et al. Nuclear Wave1 is required for reprogramming transcription in oocytes and for normal development. Science. 2013;341(6149):1002–1005. doi: 10.1126/science.1240376 23990560 PMC3824084

[85] Shindo T, Ihashi S, Sakamoto Y, et al. Visualization of endogenous nuclear F-actin in mouse embryos reveals abnormal actin assembly after somatic cell nuclear transfer. J Biochem. 2021;169(3):303–311. doi: 10.1093/jb/mvaa125 33169144

[86] Baarlink C, Wang H, Grosse R. Nuclear actin network assembly by formins regulates the SRF coactivator MAL. Science. 2013;340(6134):864–867. doi: 10.1126/science.1235038 23558171

[87] Wang Y, Sherrard A, Zhao B, et al. GPCR-induced calcium transients trigger nuclear actin assembly for chromatin dynamics. Nat Commun. 2019;10(1):5271. doi: 10.1038/s41467-019-13322-y 31754104 PMC6872576

[88] Safaralizade M, Fuderer R, Grosse R, et al. Measuring nuclear calcium and actin assembly in living cells. J Biochem. 2021;169(3):287–294. doi: 10.1093/jb/mvab002 33479753

[89] Wei M, Fan X, Ding M, et al. Nuclear actin regulates inducible transcription by enhancing RNA polymerase II clustering. Sci Adv. 2020;6(16):eaay6515. doi: 10.1126/sciadv.aay6515 32494599 PMC7159918

[90] Lundquist MR, Storaska A, Liu T-C, et al. Redox modification of nuclear actin by MICAL-2 regulates SRF signaling. Cell. 2014;156(3):563–576. doi: 10.1016/j.cell.2013.12.035 24440334 PMC4384661

[91] Chatzifrangkeskou M, Pefani D-E, Eyres M, et al. RASSF1A is required for the maintenance of nuclear actin levels. EMBO J. 2019;38(16):e101168. doi: 10.15252/embj.2018101168 31414556 PMC6694222

[92] Serebryannyy LA, Parilla M, Annibale P, et al. Persistent nuclear actin filaments inhibit transcription by RNA polymerase II. J Cell Sci. 2016;129(18):3412–25. doi: 10.1242/jcs.195867 27505898 PMC5047679

[93] Serebryannyy LA, Yuen M, Parilla M, et al. The effects of disease models of nuclear actin polymerization on the nucleus. Front Physiol. 2016;7:454. doi: 10.3389/fphys.2016.00454 27774069 PMC5053997

[94] Tsopoulidis N, Kaw S, Laketa V, et al. T cell receptor–triggered nuclear actin network formation drives CD4 + T cell effector functions. Sci Immunol. 2019;4(31):4(31. doi: 10.1126/sciimmunol.aav1987 30610013

[95] Yamazaki S, Yamamoto K, de Lanerolle P, et al. Nuclear F-actin enhances the transcriptional activity of β-catenin by increasing its nuclear localization and binding to chromatin. Histochem Cell Biol. 2016;145(4):389–399. doi: 10.1007/s00418-016-1416-9 26900020

[96] Rando OJ, Zhao K, Janmey P, et al. Phosphatidylinositol-dependent actin filament binding by the SWI/SNF-like BAF chromatin remodeling complex. Proc Natl Acad Sci U S A. 2002;99(5):2824–9. doi: 10.1073/pnas.032662899 11880634 PMC122432

[97] Chang L, Azzolin L, Di Biagio D, et al. The SWI/SNF complex is a mechanoregulated inhibitor of YAP and TAZ. Nature. 2018;563(7730):265–269. doi: 10.1038/s41586-018-0658-1 30401838 PMC7612964

[98] Huang Q, Wu D, Zhao J, et al. TFAM loss induces nuclear actin assembly upon mDia2 malonylation to promote liver cancer metastasis. EMBO J. 2022;41(11):e110324. doi: 10.15252/embj.2021110324 35451091 PMC9156967

[99] Wang A, Kolhe JA, Gioacchini N, et al. Mechanism of long-range chromosome motion triggered by gene activation. Dev Cell. 2020;52(3):309–320 e5. doi: 10.1016/j.devcel.2019.12.007 31902656 PMC7108666

[100] Chuang CH, Carpenter AE, Fuchsova B, et al. Long-range directional movement of an interphase chromosome site. Curr Biol. 2006;16(8):825–31. doi: 10.1016/j.cub.2006.03.059 16631592

[101] Dundr M, Ospina JK, Sung M-H, et al. Actin-dependent intranuclear repositioning of an active gene locus in vivo. J Cell Bio. 2007;179(6):1095–103. doi: 10.1083/jcb.200710058 18070915 PMC2140015

[102] Khanna N, Hu Y, Belmont AS. HSP70 transgene directed motion to nuclear speckles facilitates heat shock activation. Curr Biol. 2014;24(10):1138–44. doi: 10.1016/j.cub.2014.03.053 24794297 PMC4030642

[103] Ueyama H, Nakayasu H, Ueda K. Nuclear actin and transport of RNA. Cell Biol Int Rep. 1987;11(9):671–677. doi: 10.1016/0309-1651(87)90102-0 2445496

[104] Schroder HC, Trölltsch D, WENGER R, et al. Cytochalasin B selectively releases ovalbumin mRNA precursors but not the mature ovalbumin mRNA from hen oviduct nuclear matrix. Eur J Biochem. 1987;167(2):239–45. doi: 10.1111/j.1432-1033.1987.tb13329.x 3650154

[105] Lee YJ, Sheu TJ, Keng PC. Enhancement of radiosensitivity in H1299 cancer cells by actin-associated protein cofilin. Biochem Biophys Res Commun. 2005;335(2):286–91. doi: 10.1016/j.bbrc.2005.07.073 16061204

[106] Andrin C, McDonald D, Attwood KM, et al. A requirement for polymerized actin in DNA double-strand break repair. Nucleus. 2012;3(4):384–95. doi: 10.4161/nucl.21055 22688650

[107] Belin BJ, Lee T, Mullins RD. *DNA damage induces nuclear actin filament assembly by formin -2 and spire-(1/2) that promotes efficient DNA repair*. *[corrected]*. Elife. 2015;4:e07735. doi: 10.7554/eLife.07735 26287480 PMC4577826

[108] Sun MH, Yang M, Xie F-Y, et al. DNA double-strand breaks induce the nuclear actin filaments formation in Cumulus-Enclosed Oocytes but not in denuded Oocytes. PloS One. 2017;12(1):e0170308. doi: 10.1371/journal.pone.0170308 28099474 PMC5242499

[109] Caridi CP, D’Agostino C, Ryu T, et al. Nuclear F-actin and myosins drive relocalization of heterochromatic breaks. Nature. 2018;559(7712):54–60. doi: 10.1038/s41586-018-0242-8 29925946 PMC6051730

[110] Schrank BR, Aparicio T, Li Y, et al. Nuclear ARP2/3 drives DNA break clustering for homology-directed repair. Nature. 2018;559(7712):61–66. doi: 10.1038/s41586-018-0237-5 29925947 PMC6145447

[111] Lamm N, Read MN, Nobis M, et al. Nuclear F-actin counteracts nuclear deformation and promotes fork repair during replication stress. Nat Cell Biol. 2020;22(12):1460–1470. doi: 10.1038/s41556-020-00605-6 33257806

[112] Cobb AM, De Silva SA, Hayward R, et al. Filamentous nuclear actin regulation of PML NBs during the DNA damage response is deregulated by prelamin a. Cell Death Dis. 2022;13(12):1042. doi: 10.1038/s41419-022-05491-4 36522328 PMC9755150

[113] Debaugnies M, Rodríguez-Acebes S, Blondeau J, et al. RHOJ controls EMT-associated resistance to chemotherapy. Nature. 2023;616(7955):168–175. doi: 10.1038/s41586-023-05838-7 36949199 PMC10076223

[114] Palumbieri MD, Merigliano C, Gonzalez-Acosta D, et al. Nuclear actin polymerization rapidly mediates replication fork remodeling upon stress by limiting PrimPol activity. Nat Commun. 2023;14(1) 7819. doi: 10.1038/s41467-023-43183-5 38016948 PMC10684888

